# Supplementary figures and images for: Identification, Phylogeny, Divergence, Structure, and Expression Analysis of A20/AN1 Zinc Finger Domain Containing Stress-Associated Proteins (SAPs) Genes in Jatropha curcas L
Source: Genes (Basel). 2022 Sep 30;13(10):1766. doi: 10.3390/genes13101766 (PMC9601316; doi:10.3390/genes13101766)

# Ka/Ks annotated evolutionary tree

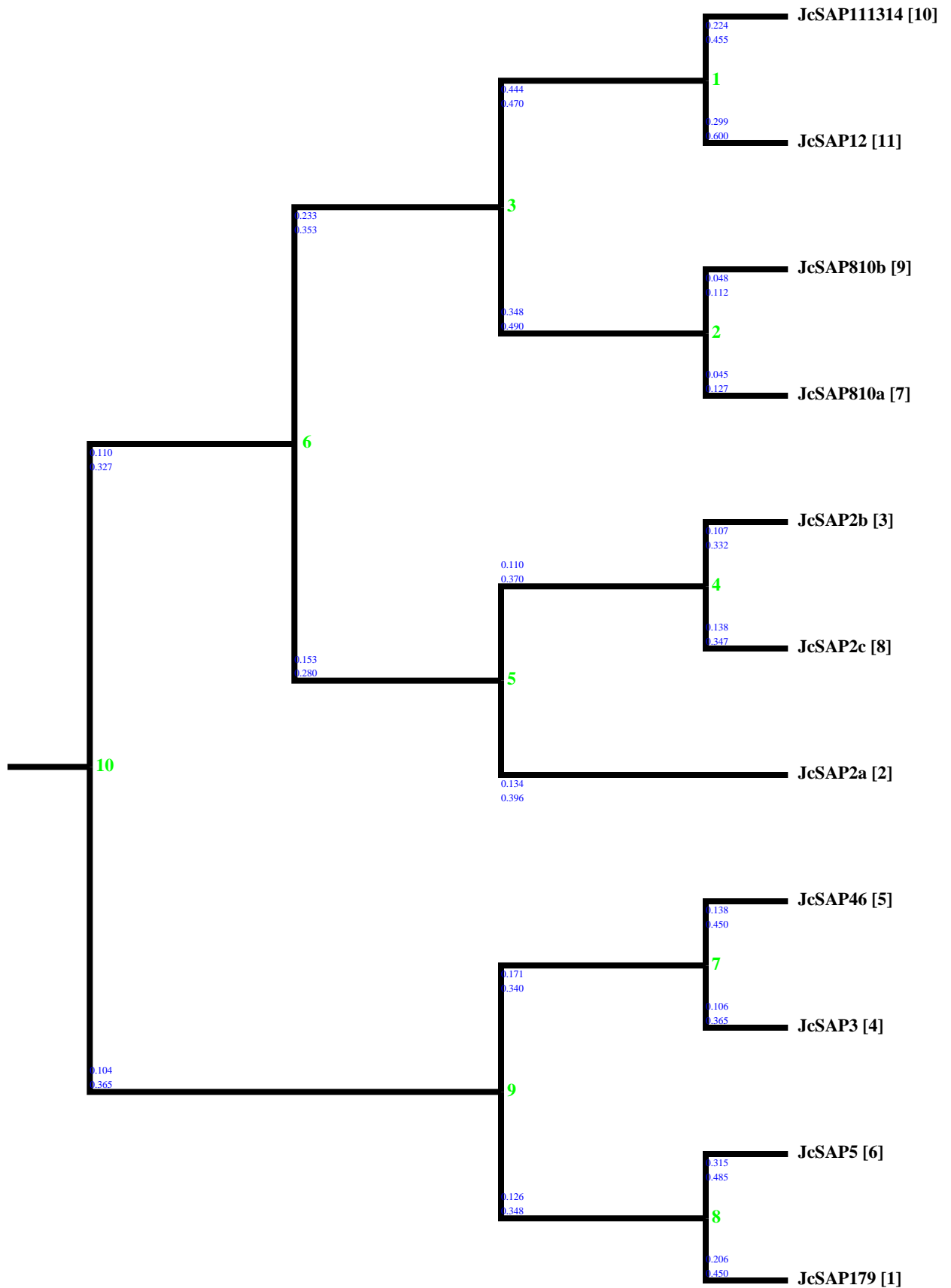

Supplement: Supplementary file 1 [file genes-13-01766-s001.zip › Figure S1.pdf]
